# Foresight beyond the very next event: four-year-olds can link past and deferred future episodes

**DOI:** 10.3389/fpsyg.2013.00404

**Published:** 2013-07-09

**Authors:** Jonathan Redshaw, Thomas Suddendorf

**Affiliations:** School of Psychology, University of Queensland, St. LuciaQLD, Australia

**Keywords:** mental time travel, episodic foresight, episodic memory, planning, problem solving

## Abstract

Previous experiments have demonstrated that by 4 years of age children can use information from a past episode to solve a problem for the very next future episode. However, it remained unclear whether 4-year-olds can similarly use such information to solve a problem for a more removed future episode that is not of immediate concern. In the current study we introduced 4-year-olds to problems in one room before taking them to another room and distracting them for 15 min. The children were then offered a choice of items to place into a bucket that was to be taken back to the first room when a 5-min sand-timer had completed a cycle. Across two conceptually distinct domains, the children placed the item that could solve the deferred future problem above chance level. This result demonstrates that by 48 months many children can recall a problem from the past and act in the present to solve that problem for a deferred future episode. We discuss implications for theories about the nature of episodic foresight.

## Introduction

Much of human cognition involves mental time travel to past and future episodes (Buckner and Carroll, 2007; Smallwood et al., 2011), a capacity that allows effective preparation for and active shaping of future events (Suddendorf and Corballis, 1997). At their core, both episodic memory and episodic foresight require self-projection into a non-current perspective (e.g., Buckner and Carroll, 2007; Suddendorf and Corballis, 2007), and one might therefore expect these abilities to draw on similar neurocognitive resources and develop at a similar age. Supporting the case of neurological similarity, both abilities engage similar brain networks (Okuda et al., 2003; Addis et al., 2007; Spreng et al., 2009; Spreng and Grady, 2010); both show similar declines in old age (Addis et al., 2008); and both are selectively impaired in hippocampus-lesioned amnesiacs (Tulving, 1985; Klein et al., 2002; Hassabis et al., 2007; Rosenbaum et al., 2009). And indeed, children who can accurately report events from yesterday are more likely to accurately report events that will happen tomorrow (Busby and Suddendorf, 2005; Suddendorf, 2010; Hayne et al., 2011). These commonalities support the view of episodic memory as a crucial design feature of the episodic foresight system (Suddendorf and Busby, 2005). Episodic memories provide a rich database of information that can be mentally recombined into similar and even entirely novel future episodes to guide prudent behavior (Schacter and Addis, 2007; Suddendorf and Corballis, 2007). Only recently, however, have studies begun to examine when and how this capacity to link past, present and future develops in children.

The ability to use information from a past episode to prepare in the present for a future episode is a useful test of mental time travel in both non-verbal animals and children whose verbal responses may belie their true cognitive abilities (Hampton and Schwartz, 2004; Suddendorf and Busby, 2005; Tulving, 2005; Scarf et al., 2013). Suddendorf and Corballis (2010) distil four criteria that studies of this capacity must meet in order to rule out alternative explanations: (1) use of single trials, to avoid conditioning and demonstrate memory for a specific event; (2) use of novel problems, to engage cognitive processes and eliminate learning history explanations; (3) use of separate spatial/temporal contexts for the crucial future-directed action and the future problem, to avoid cuing to the answer and demonstrate long-term memory; and (4) use of multiple problems from distinct domains, to demonstrate the domain-general nature of the capacity. Compelling positive evidence meeting these criteria has not yet been obtained for animals (Suddendorf and Corballis, 2010), and some studies of children also fail to meet them. In a recent study by Russell and colleagues (2010), for example, the future problem was presently visible to the children during the future-directed action (violating criterion 3), such that the successful children did not necessarily have to base their solution on a spatially and temporally removed mental construction.

Suddendorf and colleagues (2011) designed a series of experiments that did meet the four criteria outlined above. In one of these, 3- and 4- year-olds were introduced to one of two conceptually distinct problems in an initial testing room. The “box task” involved a box with a triangle-shaped keyhole and the “food task” involved a puppet that wanted to eat a banana. In the instant condition, the child was led to the other side of the room immediately after being presented with the problem, and was allowed to choose a solution from a selection of three options without being allowed to look back at the problem. In the delay condition, the child was taken to a second room and distracted for 15 min. They were then offered the same choice for an immediate return to the first room. Importantly, the experimenter did not refer to the problem in either condition, only its location. In the instant condition, both 3- and 4-year-olds selected the item that could solve the problem at levels close to ceiling. In the delay condition, however, only the 4-year-olds selected the item that secured the future solution significantly above chance level.

These results clearly demonstrate the effect of a delay between the relevant past event and the future-oriented action on children's ability to link past and future episodes. Only 4-year-olds demonstrated a capacity to import a past event from long-term memory into working memory and act for the future. Still, the very next event following item selection was a return to the problem, and so, while the future problem had to be imagined, in some sense it may have been part of the child's psychological present. Even 24-month-old children (Bauer et al., 1999) and non-human great apes (Koehler, 1927; Döhl, 1968) show some ability to mentally simulate and act for the “future,” when that future is part of a single ongoing event and long-term memory is not required. This ability likely relies on working memory in much the same way as the ability to recall and manipulate information that has been encountered in the very recent past. As adults, however, we frequently prepare for more remote future events, and we rely on our long-term memory for what was required in similar past episodes to guide our future-oriented actions. It is possible that imagining and acting for a deferred future episode requires more executive resources than for a future episode that has become of immediate concern, such as the episode in Suddendorf and colleagues' (2011) task. Would 4-year-olds be capable of solving this task if a series of irrelevant events was inserted between the future-directed action and the return to the original problem? Can they link past and future episodes that are *both* temporally removed from the immediate?

Attempts to test for episodic foresight beyond the very next event must overcome the difficult problem of how to inform a child participant just when in the future their present behavior will have an effect (Suddendorf et al., 2011). Young children, especially those aged four and under, have considerable trouble understanding and correctly using specific future-oriented terms (Harner, 1975, 1980; Busby Grant and Suddendorf, 2010, 2011). Therefore, informing them that their choice of an item will have an effect when they go to another room in “five minutes” or play a game “tomorrow” (e.g., Russell et al., 2010) would be futile for a large proportion of young children. However, as children generally understand concrete instantiations of concepts earlier than they understand symbolic representations (Uttal et al., 1997), it may be more effective to convey the delay with a sand-timer. A previous study revealed that the majority of 4-year-olds understand that a half empty sand-timer will complete its cycle before a full one (Bischof-Köhler, 2000), suggesting that they correctly recognize something about how the amount of sand in the compartments changes over time.

The current experiment built on Suddendorf and colleagues' (2011) methodology to test whether 4-year-olds could remember a problematic past episode from 15 min ago and prepare for a deferred future episode marked by the completion of a sand-timer's cycle. Rather than choosing an item to immediately take into the problem room, the children instead had to place the item into a bucket, which they were previously told would be taken to the problem room only when the timer had completed its cycle. The inclusion of the bucket crucially required the children to physically separate themselves from their chosen item, thus reinforcing the idea that the item was to be “saved” for a deferred future episode rather than used in the immediate future. An instant condition with no delay in either temporal direction was included to confirm that the problems themselves were conceptually easy.

## Method

### Participants

Twenty-four 4-year-olds (*M* = 48 months 17 days, *SD* = 14 days) were included in the study. This group consisted of 10 boys and 14 girls, who each participated individually with a parent or caregiver present. Each child completed both the box task and the food task, one in the instant condition and one in the delayed condition. The orders of the tasks and conditions were counterbalanced.

### Materials

The experimental materials for the box task and the food task were identical to those used in experiment two of Suddendorf and colleagues' (2011) study, with the exception that more distracting objects were included to lower the level of chance performance and thus increase experimental power.

#### Box task

Two wooden boxes (14 × 21 × 21 cm) were used, each featuring a large keyhole (either square- or triangle-shaped). Sliding an appropriately-shaped key into these keyholes activated a mechanism that revealed a previously hidden platform within the box, allowing objects to be retrieved from this platform. Seven different keys were used, each consisting of a shape connected to a 19 cm rod. The seven shapes were: square, triangle, circle, star, heart, teardrop, and an irregular zigzag shape.

#### Food task

Two commercially available hand puppets (a tiger and an elephant) were used, along with seven plastic foods: strawberry, banana, apple, pear, orange, grapes, and carrot.

#### Sand-timer and bucket

One commercially available cylindrical sand-timer (height 16 cm, diameter 8 cm) with blue sand grains was used. The time of a complete cycle was approximately 5 min. A black bucket was also used, in which the child was to place their selected item in the delayed condition.

### Procedure

Prior to the main sequence of the experiment, the child was taken to a warm-up room where they were introduced to the time-keeping nature of the sand-timer. The sand-timer was turned over and the child was asked to examine the sand falling from the top to the bottom compartment. The child was informed that they would receive a sticker once all of the sand had reached the bottom.

After this introduction, the child was taken to Room A, referred to as “Charlie the chicken's room” owing to a large poster of a chicken on the wall. In this room they were presented with the box task and the food task while seated at a child-friendly table.

#### Box task

The experimenter introduced the child to the box with the square-shaped hole, and demonstrated how to use the square-shaped key to activate the mechanism and reveal a hidden toy. The child was allowed to perform this action themselves, before the demonstration was repeated and the child was allowed to have a second attempt. This box was then removed and the box with the triangle-shaped hole was introduced. The experimenter demonstrated to the child that the square-shaped key did not work on this box, and the child was allowed to confirm this for themselves.

In the instant condition, the child was then led to the other side of the room, containing a table with the triangle-shaped key and five distracter keys. Without being able to look back at the test table, the child was asked to pick one of these items to take back.

In the delayed condition, the child was told that they would go to another room (Room B) to play some games, and the box with triangle-shaped keyhole was left on the table. After 15 min of unrelated activity in Room B, the child was reintroduced to the sand-timer and shown the bucket. They were informed that (1) they would be going back to Charlie the chicken's room after the sand-timer had completed a cycle, and (2) they would be taking the bucket with them when they went. The child was asked to independently generate these facts consecutively to ensure that they understood exactly what would be happening and when. Finally, the experimenter revealed a previously hidden tray containing the triangle-shaped key and the five distracter keys and invited the child to place one of these items only into the bucket. After they had made their choice, the child was asked to explain the reason for their selection. Upon completion of the sand-timer's cycle, the child was invited to bring the bucket with them to Room A where they could solve the problem (the experimenter brought the correct item if the child had not selected it).

#### Food task

The experimenter introduced the child to the tiger puppet. The child was told that “Terry the tiger” liked to eat strawberries and the child was allowed to “feed” the puppet using the visible plastic strawberry on the table. This procedure was repeated, before the child was introduced to the elephant puppet and told that “Ellie the elephant” liked to eat bananas. The experimenter then pointed out that there were no bananas to feed Ellie.

The instant and delayed conditions mirrored those of the box task, except that the items available to choose were the plastic banana and the five plastic food distracters.

## Results

A 2 × 2 ANOVA (Task × Condition) revealed a main effect of Condition, with the children selecting the correct item significantly more often in the instant condition than the delayed condition, *F*_(1, 22)_ = 12.79, *p* = 0.002. There were no significant effects involving Task, suggesting that the box and food tasks did not vary in difficulty in either condition. There were also no significant condition order effects, suggesting that the presentation order of the instant and delayed conditions did not affect the children's performance. We have therefore collapsed across these variables in the subsequent analyses.

**Figure 1:**
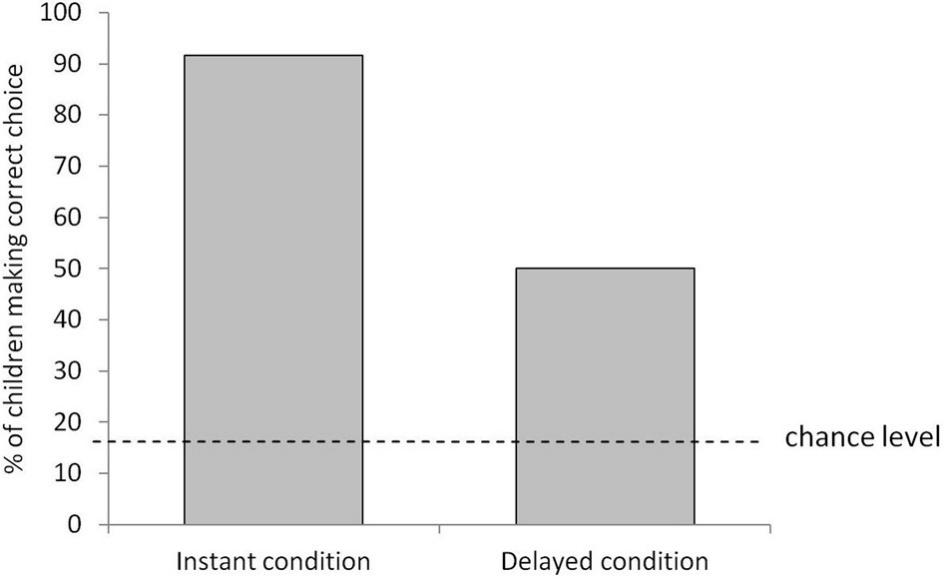
**Proportion of 4-year-olds who made the correct item choice in the instant and delayed conditions (collapsed across the box and food tasks)**.

As seen in Figure 1, the large majority of children selected the appropriate item (91.7%) in the instant condition, a performance that was well above chance level (16.7%), χ^2^(1) = 97.20, *p* < 0.001. This finding confirmed that the problems themselves were conceptually easy when the temporal element was absent. Although performance was decreased in the delayed condition, the children still selected the appropriate item (50%) well above chance level, χ^2^(1) = 19.20, *p* < 0.001.

The conclusion that the children displayed some foresight when selecting the item in the delayed condition is supported by their verbal responses. Across tasks, 25% of children made reference to the future utility of the item when explaining their choice. All of these children had selected the correct item, a performance that was well above chance level, χ^2^(1) = 30.00, *p* < 0.001. Of the 75% of children that did not make future reference when explaining their choice, one third of them had still selected the correct item. Nevertheless, the rate of correct item selection among this sub-sample was not significantly above chance level, χ^2^(1) = 3.60, *p* = 0.058.

## Discussion

The current study examined whether 4-year-olds could remember a problem from a specific past episode and act in the present to obtain a novel solution for a return to the problem in a deferred future episode. In the delayed condition, the children had to independently confirm their understanding that they would be returning to Room A with the bucket after the familiar sand-timer had completed its cycle. This process ensured that the children understood the item they subsequently placed into the bucket was not to be used in the immediate future, but rather saved for the deferred future episode in Room A. And, although they did not perform at the same level as in the instant condition, they still selected the correct item well above chance level. The current study therefore demonstrates, for the first time, that many 4-year-olds can use information from past episodes to prepare for deferred future episodes, just as they can for otherwise identical future episodes of immediate concern (Suddendorf et al., 2011).

The findings of the current study add to the growing developmental literature that has emerged following recent widespread recognition of the importance of human episodic foresight and its links to episodic memory (Science, 2007). Developmental milestones appear to be achieved over the preschool years (Suddendorf and Moore, 2011; Suddendorf and Redshaw, in press). During this period, children begin to show competence on tasks requiring them to delay gratification for the future (Mischel et al., 1989; Moore et al., 1998; Garon et al., 2012), save resources for the future (Atance and Meltzoff, 2005; Metcalf and Atance, 2011), place multiple future episodes in time relative to each other (Friedman, 2000; Busby Grant and Suddendorf, 2009; Hayne et al., 2011; Hudson and Mayhew, 2011), learn a rule to be applied in the future (Kliegel and Jäger, 2007), plan an intervention that will help a character in the future (McColgan and Mccormack, 2008; McCormack and Hanley, 2011), and evaluate the likelihood of future events (Lagattuta and Sayfan, 2011). The current study documents the development of another key future-oriented capacity: adaptively linking past and deferred future episodes.

### Potential implications for the nature of episodic foresight

The performance of the 4-year-olds in the delayed condition (50% success, with a chance level of 16.7%) closely matches that of the 4-year-olds in Suddendorf and colleagues' (2011) second experiment, in which the future episode immediately followed the crucial future-oriented action (58.3% success, with a chance level of 33.3%). Perhaps then, as long as the relevant past episode can be imported into working memory, we can use the same cognitive mechanism to prepare for a similar future episode whether that future episode is the very next event or several minutes removed. So, as long as we can remember the shampoo bottle running out last night, we can use the same mechanism to replace it with a full one whether we are going to shower immediately or in preparation for a shower in 5 min time. This observation raises the possibility that the same mechanism can be used to prepare for even more distant future episodes, such as next summer's holiday, again as long as the relevant past episode is recalled.^1^ Such an interpretation is consistent with the strong neurological links between episodic memory and episodic foresight described in the introduction, and with the idea that episodic memory may have evolved as a crucial design feature of the episodic foresight system.

If imagining immediate and deferred future episodes can be achieved with the same basic mechanism, then it may follow that children (and adults) will tend to use this mechanism as a heuristic when preparing for any deferred future episode—even when additional mechanisms *are* required. Consistent with Read and Loewenstein's(1995) time contraction hypothesis, we may typically imagine and prepare for any future episode as if it were occurring in the immediate future; as if the current self were placed into that episode with a time machine. Nevertheless, our current motivation, emotion, and knowledge states are not always the same as our future states, and so we must sometimes prepare for a deferred future episode containing a self that differs from the current self on some important dimension (Suddendorf and Corballis, 1997). Both children (Atance and Meltzoff, 2006) and adults (Nisbett and Kanouse, 1969) often fail to take this difference into account when required, despite the fact that 4-year-olds are beginning to demonstrate a capacity to imagine minds with distinct states to their own (Wellman et al., 2001). These specific *theory-of-future-mind* failings may be explained by the routine use of a generally successful heuristic that places the current self into the future episode.

### Future directions and conclusions

The current study was designed only to answer the specific question of whether 4-year-olds could prepare for a deferred future event just as they can for the very next future event, and we found that indeed they could. Future studies may want to vary the temporal delay between the item selection and the future event. It is possible that the same basic mechanism can be used to prepare for an immediate future event and one that is happening in 5 min, but additional mechanisms are required to prepare for an event happening next year. Such a finding would provide evidence against the heuristic view we have presented above, which theoretically should apply to any non-immediate future event. Other studies may want to vary the temporal period in which an item becomes useful, such that the item that is useful immediately is not the same one that is useful in 5 min or in 10 min. Selecting only the time-appropriate item may require complex inhibition skills, which 4-year-olds are still in the process of learning (Gerstadt et al., 1994; Diamond and Taylor, 1998).

In conclusion, the current study has demonstrated, for the first time, that 4-year-olds can remember a problem from a specific past episode and act to solve that problem for a deferred future episode, just as they can for the very next future episode. Future-oriented action may be achievable with the same episodic foresight mechanism whether or not intermediate events are located between that action and the relevant future episode.

### Conflict of interest statement

The authors declare that the research was conducted in the absence of any commercial or financial relationships that could be construed as a potential conflict of interest.

## Footnotes

^1^This is not to say we are equally motivated to prepare for immediate and distant future events, as a large body of temporal discounting literature disproves (e.g., Green et al., 1994; Green and Myerson, 2004). Future events are increasingly uncertain as they move further away, and so our lack of enthusiasm for preparing for distant episodes is sometimes justified. Still, the current study may indeed suggest that linking past events with deferred future events is not more cognitively demanding in principle than for immediate future events.

## References

[B1] Addis D. R. Wong A. T. Schacter D. L. (2007). Remembering the past and imagining the future: Common and distinct neural substrates during event construction and elaboration. Neuropsychologia 45, 1363–1377 10.1016/j.neuropsychologia.2006.10.016 17126370 PMC1894691

[B2] Addis D. R. Wong A. T. Schacter D. L. (2008). Age-related changes in the episodic simulation of future events. Psychol. Sci. 19, 33–41 10.1111/j.1467-9280.2008.02043.x 18181789

[B3] Atance C. M. Meltzoff A. N. (2005). My future self: Young children's ability to anticipate and explain future states. Cognitive Dev. 20, 341–361 10.1016/j.cogdev.2005.05.001 PMC3744374 23956493

[B4] Atance C. M. Meltzoff A. N. (2006). Preschoolers' current desires warp their choices for the future. Psychol. Sci. 17, 583–587 10.1111/j.1467-9280.2006.01748.x 16866743 PMC1523428

[B5] Bauer P. J. Schwade J. A. Wewerka S. S. Delaney K. (1999). Planning ahead: goal-directed problem solving by 2-year-olds. Dev. Psychol. 35, 1321–1337 10.1037/0012-1649.35.5.1321 10493657

[B6] Bischof-Köhler D. (2000). Kinder auf Zeitreise: Theory of Mind, Zeitverständnis und Handlungsorganisation [Children in time: Theory of mind, time understanding and action organisation]. Bern: Hogrefe & Huber.

[B7] Buckner R. L. Carroll D. C. (2007). Self-projection and the brain. Trends Cogn. Sci. 11, 49–57 10.1016/j.tics.2006.11.004 17188554

[B8] Busby Grant J. Suddendorf T. (2009). Preschoolers begin to differentiate the times of events from throughout the lifespan. Eur. J. Dev. Psychol. 6, 746–762 10.1080/17405620802102947

[B9] Busby Grant J. Suddendorf T. (2010). Young children's ability to distinguish past and future changes in physical and mental states. Br. J. Dev. Psychol. 28, 853–870 10.1348/026151009X482930 21121471

[B10] Busby Grant J. Suddendorf T. (2011). Production of temporal terms by 3-, 4-, and 5-year-old children. Early Child Res. Q. 26, 87–95 10.1016/j.ecresq.2010.05.002

[B11] Busby J. G. Suddendorf T. (2005). Recalling yesterday and predicting tomorrow. Cogn. Dev. 20, 362–372 10.1016/j.cogdev.2005.05.002

[B12] Diamond A. Taylor C. (1998). Development of an aspect of executive control: development of the abilities to remember what I said and to “Do as I say, not as I do”. Dev. Psychobiol. 29, 315–334 8732806 10.1002/(SICI)1098-2302(199605)29:4<315::AID-DEV2>3.0.CO;2-T

[B13] Döhl J. (1968). Versuche mit einer Schimpansin uber Abkurzungen bei Umwegen mit selbststandigen Zwischenzielen [Experiments with a chimpanzee on using shortcuts in detour problems with subgoals]. Z. Tierpsychol. 26, 200–207 10.1111/j.1439-0310.1969.tb01945.x

[B14] Friedman W. J. (2000). The development of children's knowledge of the times of future events. Child Dev. 71, 913–932 10.1111/1467-8624.00199 11016556

[B15] Garon N. M. Longard J. Bryson S. E. Moore C. (2012). Making decisions about now and later: development of future-oriented self-control. Cogn. Dev. 27, 314–322 10.1016/j.cogdev.2012.05.003

[B16] Gerstadt C. L. Hong Y. J. Diamond A. (1994). The relationship between cognition and action: performance of children 3 1/2 – 7 years old on a stroop-like day-night test. Cognition 53, 129–153 10.1016/0010-0277(94)90068-X 7805351

[B17] Green L. Fry A. F. Myerson J. (1994). Discounting of delayed rewards: a life-span comparison. Psychol. Sci. 5, 33–36 10.1111/j.1467-9280.1994.tb00610.x

[B18] Green L. Myerson J. (2004). A discounting framework for choice with delayed and probabilistic rewards. Psychol. Bull. 130, 769–792 10.1037/0033-2909.130.5.769 15367080 PMC1382186

[B19] Hampton R. R. Schwartz B. L. (2004). Episodic memory in nonhumans: what, and where, is when? Curr. Opin. Neurobiol. 14, 192–197 10.1016/j.conb.2004.03.006 15082324

[B20] Harner L. (1975). Yesterday and tomorrow: development of early understanding of the terms. Dev. Psychol. 11, 864–865 10.1037/0012-1649.11.6.864

[B21] Harner L. (1980). Comprehension of past and future reference revisited. J. Exp. Child Psychol. 29, 170–182 10.1016/0022-0965(80)90099-5 7354264

[B22] Hassabis D. Kumaran D. Vann S. D. Maguire E. A. (2007). Patients with hippocampal amnesia cannot imagine new experiences. Proc. Natl. Acad. Sci. U.S.A. 104, 1726–1731 10.1073/pnas.0610561104 17229836 PMC1773058

[B23] Hayne H. Gross J. McNamee S. Fitzgibbon O. Tustin K. (2011). Episodic memory and episodic foresight in 3-and 5-year-old children. Cogn. Dev. 26, 343–355 10.1016/j.cogdev.2011.09.006

[B24] Hudson J. A. Mayhew E. M. Y. (2011). Children's temporal judgments for autobiographical past and future events. Cogn. Dev. 26, 331–342 10.1016/j.cogdev.2011.09.005

[B25] Klein S. B. Loftus J. Kihlstrom J. F. (2002). Memory and temporal experience: the effects of episodic memory loss on an amnesic patient's ability to remember the past and imagine the future. Soc. Cogn. 20, 353–379 10.1521/soco.20.5.353.21125

[B26] Kliegel M. Jäger T. (2007). The effects of age and cue-action reminders on event-based prospective memory performance in preschoolers. Cogn. Dev. 22, 33–46 10.1016/j.cogdev.2006.08.003

[B27] Koehler W. (1927). The Mentality of Apes. London: Routledge & Kegan Paul Ltd 10.1037/11338-000

[B28] Lagattuta K. H. Sayfan L. (2011). Developmental changes in children's understanding of future likelihood and uncertainty. Cogn. Dev. 26, 315–330 10.1016/j.cogdev.2011.09.00412708613

[B29] McColgan K. L. Mccormack T. (2008). Searching and planning: young children's reasoning about past and future event sequences. Child Dev. 79, 1477–1497 10.1111/j.1467-8624.2008.01200.x 18826537

[B30] McCormack T. Hanley M. (2011). Children's reasoning about the temporal order of past and future events. Cogn. Dev. 26, 299–314 10.1111/j.1467-8624.2008.01200.x 18826537

[B31] Metcalf J. L. Atance C. M. (2011). Do preschoolers save to benefit their future selves? Cogn. Dev. 26, 371–382 10.1016/j.cogdev.2011.09.003

[B32] Mischel W. Shoda Y. Rodriguez M. I. (1989). Delay of gratification in children. Science 244, 933–938 10.1126/science.2658056 2658056

[B33] Moore C. Barresi J. Thompson C. (1998). The cognitive basis of future-oriented prosocial behavior. Soc. Dev. 7, 198–218 10.1111/1467-9507.00062

[B34] Nisbett R. E. Kanouse D. E. (1969). Obesity, food deprivation, and supermarket shopping behavior. J. Pers. Soc. Psychol. 12, 289–294 10.1037/h0027799 5821855

[B35] Okuda J. Fujii T. Ohtake H. Tsukiura T. Tanji K. Suzuki K. (2003). Thinking of the future and past: the roles of the frontal pole and the medial temporal lobes. Neuroimage 19, 1369–1380 10.1016/S1053-8119(03)00179-4 12948695

[B36] Read D. Loewenstein G. (1995). Diversification bias: explaining the discrepancy in variety seeking between combined and separated choices. J. Exp. Psychol. Appl. 1, 34–49 10.1037/1076-898X.1.1.34

[B37] Rosenbaum R. S. Gilboa A. Levine B. Winocur G. Moscovitch M. (2009). Amnesia as an impairment of detail generation and binding: evidence from personal, fictional, and semantic narratives in K. C. Neuropsychologia 47, 2181–2187 10.1016/j.neuropsychologia.2008.11.028 19100757

[B38] Russell J. Alexis D. Clayton N. (2010). Episodic future thinking in 3-to 5-year-old children: the ability to think of what will be needed from a different point of view. Cognition 114, 56–71 10.1016/j.cognition.2009.08.013 19781693

[B39] Scarf D. Gross J. Colombo M. Hayne H. (2013). To have and to hold: episodic memory in 3-and 4-year-old children. Dev. Psychobiol. 55, 125–132 10.1002/dev.21004 22213009

[B40] Schacter D. L. Addis D. R. (2007). The cognitive neuroscience of constructive memory: remembering the past and imagining the future. Philos. Trans. R. Soc. Lond. B Biol. Sci. 362, 773–786 10.1098/rstb.2007.2087 17395575 PMC2429996

[B41] Science. (2007). Breakthrough of the year: the runners-up. Science 318, 1844–1869 10.1126/science.318.5858.1844a 18096772

[B42] Smallwood J. Schooler J. W. Turk D. J. Cunningham S. J. Burns P. Macrae C. N. (2011). Self-reflection and the temporal focus of the wandering mind. Conscious. Cogn. 20, 1120–1126 10.1016/j.concog.2010.12.017 21277803

[B43] Spreng R. N. Grady C. L. (2010). Patterns of brain activity supporting autobiographical memory, prospection, and theory of mind, and their relationship to the default mode network. J. Cogn. Neurosci. 22, 1112–1123 10.1162/jocn.2009.21282 19580387

[B44] Spreng R. N. Mar R. A. Kim A. S. N. (2009). The common neural basis of autobiographical memory, prospection, navigation, theory of mind, and the default mode: a quantitative meta-analysis. J. Cogn. Neurosci. 21, 489–510 10.1162/jocn.2008.21029 18510452

[B46] Suddendorf T. (2010). Linking yesterday and tomorrow: Preschoolers' ability to report temporally displaced events. Br. J. Dev. Psychol. 28, 491–498 10.1348/026151009X479169 20481400

[B48] Suddendorf T. Busby J. G. (2005). Making decisions with the future in mind: developmental and comparative identification of mental time travel. Learn. Motiv. 36, 110–125 10.1016/j.lmot.2005.02.010

[B49] Suddendorf T. Corballis M. C. (1997). Mental time travel and the evolution of the human mind. Genet. Soc. Gen. Psychol. Monogr. 123, 133–167 9204544

[B50] Suddendorf T. Corballis M. C. (2007). The evolution of foresight: what is mental time travel, and is it unique to humans? Behav. Brain Sci. 30, 299–313 10.1017/S0140525X07001975 17963565

[B52] Suddendorf T. Corballis M. C. (2010). Behavioural evidence for mental time travel in nonhuman animals. Behav. Brain Res. 215, 292–298 10.1016/j.bbr.2009.11.044 19962409

[B53] Suddendorf T. Moore C. (2011). Introduction to the special issue: the development of episodic foresight. Cogn. Dev. 26, 295–298 10.1016/j.cogdev.2011.09.001

[B54] Suddendorf T. Nielsen M. Von Gehlen R. (2011). Children's capacity to remember a novel problem and to secure its future solution. Dev. Sci. 14, 26–33 10.1111/j.1467-7687.2010.00950.x 21159085

[B55] Suddendorf T. Redshaw J. (in press). The development of mental scenario building and episodic foresight. Ann. N. Y. Acad. Sci. 10.1111/nyas.12189. [Epub ahead of print]. 23855564

[B56] Tulving E. (1985). Memory and consciousness. Can. Psychol. 26, 1–12 10.1037/h0080017

[B57] Tulving E. (2005). Episodic memory and autonoesis: uniquely human?, in The Missing Link in Cognition: Origins of Self-reflective Consciousness, eds Terrace H. S. Metcalfe J. (Oxford: Oxford University Press), 3–56 10.1093/acprof:oso/9780195161564.003.0001

[B58] Uttal D. H. Scudder K. V. Deloache J. S. (1997). Manipulatives as symbols: a new perspective on the use of concrete objects to teach mathematics. J. Appl. Dev. Psychol. 18, 37–54 10.1016/S0193-3973(97)90013-7

[B59] Wellman H. M. Cross D. Watson J. (2001). Meta-analysis of theory-of-mind development: the truth about false belief. Child Dev. 72, 655–684 10.1111/1467-8624.00304 11405571

